# Systematic analysis of lysine 2-hydroxyisobutyrylation posttranslational modification in wheat leaves

**DOI:** 10.1371/journal.pone.0253325

**Published:** 2021-06-17

**Authors:** Bo Feng, Shengdong Li, Zongshuai Wang, Fang Cao, Zheng Wang, Geng Li, Kaichang Liu

**Affiliations:** 1 Crop Research Institute, Shandong Academy of Agricultural Sciences, Ji’nan, Shandong, P. R. China; 2 College of Agronomy, Shandong Agricultural University, Tai’an, Shandong, P. R. China; National Agri-Food Biotechnology Institute (NABI) Mohali, INDIA

## Abstract

Lysine 2-hydroxyisobutyrylation (Khib) is a recently discovered post-translational modification (PTM) showing diverse biological functions and effects in living organisms. However, the study of Khib in plant species is still relatively limited. Wheat (*Triticum aestivum* L.) is a global important cereal plant. In this study, the systematic Khib analysis was performed in wheat leave tissues. A total of 3004 Khib sites in 1104 proteins were repeatedly identified. Structure characterization of these Khib peptides revealed 12 conserved sequence motifs. Function classification and enrichment analysis indicated these Khib proteins showed a wide function and pathway distribution, of which ribosome activity, protein biosynthesis and photosynthesis were the preferred biological processes. Subcellular location predication indicated chloroplast was the dominant subcellular compartment where Khib was distributed. There may be some crosstalks among Khib, lysine acetylation and lysine succinylation modification because some proteins and sites were modified by all these three acylations. The present study demonstrated the critical role of Khib in wheat biological and physiology, which has expanded the scope of Khib in plant species. Our study is an available resource and reference of Khib function demonstration and structure characterization in cereal plant, as well as in plant kingdom.

## Introduction

Protein post-translational modifications (PTMs), the covalent processing events that cleaving or adding a modifying group to an amino acid or more amino acids, are essential regulatory patterns of diverse biological processes and cellular events [1]. Increasing new PTMs have been reported due to the development of high-resolution mass spectrometry (MS), such as acetylation (Kac), succinylation (Ksucc), propionylation, butyrylation and crotonylation etc [2–4]. With bioinformatics tools, their biological processes regulation and metabolic pathways adjustment roles have been illustrated to some extent [2–4]. To data, more than 400 distinct PTMs have been reported in living various organisms [5]. Lysine 2-hydroxyisobutyrylation (Khib) is a recently reported novel naturally occurring PTM that was first proved active in histone and indicated a critical role in regulating chromatin functions and gene transcription [6–8]. Moreover, it has been reported that Esa1p in yeast and its homolog Tip60 in humans promoted the addition of Khib to substrate proteins while histone deacetylase 2 (HDAC2) and HDAC3 participated in removing Khib [9].

To explore the potential regulatory role of Khib in biological processes and cellular events, lysine 2-hydroxyisobutylome has been performed in various species. In *Saccharomyces cerevisiae*, it has been reported that evolutionarily conserved histone H4K8 Khib (H4K8hib) fluctuated in response to glucose homeostasis [10]. Further proteomic analysis revealed that 1,458 Khib sites on 369 proteins were involved in glycolysis, gluconeogenesis and ribosome [10]. In bacteria, *Escherichia coli* and *Proteus mirabilis* have been studied. The screened 4735 Khib sites on 1051 proteins in *P*. *mirabilis* are involved in diverse metabolic pathways, indicating the modification may influence bacterial metabolism [11]. In *E*. *coli* cells, quantitative proteomics approach was used to characterize the effects of *CobB* gene deletion on the 2-hydroxyisobutyrylome profile of *E*. *coli* and the results indicated *CobB* regulated Khib level may influence cellular metabolism [12].

In animals, especially mammals, relatively extensive Khib related studies have been conducted. The first parasites 2-hydroxyisobutyrylome analysis has been performed in two *T*. *gondii* strains (RH and ME49). The result indicated Khib proteins were distributed in diverse subcellular locations and involved in a wide variety of biological functions and cellular processes [13]. In mammals, various cell lines were used as materials to study Khib such as HeLa cells, mouse embryonic fibroblast cells, Drosophila S2 cells, A549 cells, 5637 cells, HEK293T cells and pluripotent stem cells [6,9,11,14–16]. Qualitative 2-hydroxyisobutyrylome analysis in Hela cell identified 6,548 Khib sites in 1,725 proteins and these Khib proteins showed a diverse subcellular location distribution [9]. Two quantitative 2-hydroxyisobutyrylome analyses have been performed to reveal the role of protein Khib in some drug mediated cancer treatment; and the results have shown that the antitumor effects of some drugs are associated with changed protein Khib level [14,15].

The related research is very limited in plant and only the 2-hydroxyisobutyrylome of rice and *Physcomitrella patens* has been studied [17,18]. A total of 9,916 Khib sites in 2,512 proteins have been identified in developing rice seeds which involved in a wide variety of vital biological processes and metabolism pathways [17]. In addition, some Khib sites were shared with other lysine PTMs, including Ksucc, Kac, malonylation and crotonylation [17,19]. In *Physcomitrella*, 11,976 Khib sites in 3,001 proteins were detected [18]. Nevertheless, the study of Khib in plants is still limited, especially the involved plant species is too less.

In the present study, We aim to identify potential Khib substrates and demonstrate the action mechanism of Khib in wheat through antibody based immunoprecipitation affinity enrichment and advanced mass spectrometry based proteomic techniques.

## Materials and methods

### Plant materials

The common wheat variety (*Triticum aestivum* L.) Jimai 44 was used in this study. The wheat cultivation was performed in the experimental field of Shandong Academy of Agricultural Sciences (SAAS) in Ji’nan (36°42’ N, 117°4’ E; altitude 48 m), Shandong Province, China. The average annual amount of sunshine was 2600 h, the average annual temperature was 14.5°C, and the mean precipitation was approximately 700 mm. In 2019 wheat sowing season, the wheat seed were grown in the clay pots (diameter 30 cm, height 35 cm) filled with 20 kg soil obtained from the aforementioned experiment field, combining with a protective net to protect the seeds and seedlings from being eaten or damaged by pest, field mouse or sparrow. The wheat plants were irrigated with normal water and fertilization condition. After 40 days cultivation, wheat leaf tissues were harvested into the liquid nitrogen immediately and then stored at -80°C until for further use. All samples were collected in three biological replicates.

### Protein extraction and digestion

The protein extraction and trypsin digestion were performed referring previous report with some modifications [20]. In brief, wheat leaves were grinded into powder in a mortar with the addition of liquid nitrogen. The powder was dissolved in extraction buffer (8 M urea, 2 mM EDTA, 3 μM TSA, 50 mM NAM, 10 mM DTT and 1% protease Inhibitor Cocktail) and sonicated for 10 minutes on ice. The remaining cell debris were depleted through centrifugation at 15 000 g for 20 min at 4°C. Then the supernatant was precipitated with ice-cold acetone for 8 hour at −20°C and then centrifuged at 15 000 g for 20 min at 4°C. The obtained protein pellet was washed with cold acetone three times.

In the digestion process, the protein was dissolved in buffer (8 M urea, 100 mM NH_4_HCO_3_, pH 8.0) and quantified with a 2-D Quant kit (GE Healthcare, America) following the manufacturer’s instructions. Then the sample was incubated with 5 mM DTT at 37°C for 60 min. The alkylation reaction was conducted with 11 mM iodoacetamide for 30 min at room temperature in darkness. TEAB solution with concentration 100 mM was added into the protein sample to reduce urea concentration to less than 2 M to diminish the interference effects of urea in digestion. A two-step trypsin digestion was carried out to completely digest the proteins. Finally, peptide was desalted by Strata X C18 SPE column (Phenomenex) and vacuum-dried.

### Khib peptides enrichment

To purify the 2-hydroxyisobutyrylation modified peptides form the peptides mixture, an antibody conjugated beads (WM502, Micrometer Biotech, Hangzhou) based immunoprecipitation process was carried out. Fractionated peptides were dissolved in NETN buffer (100 mM NaCl, 1 mM EDTA, 50 mM Tris-HCl, 0.5% NP-40, pH 8.0). Then the peptides were incubated with pan anti-acetyllysine antibody beads in NETN buffer at 4°C for 16 h with gentle rotation. After incubation, rinse the beads for 4 times with NETN buffer and 2 times with purified water. Elute the captured modified peptides with 0.1% Trifluoroacetic acid and vacuum dry them [20].

### LC-MS/MS analysis

LC-MS/MS analysis was carried out following previous report [19]. The dried peptides were re-suspended in buffer A (0.1% FA, 100% H_2_O) and followed a centrifugation process. The supernatant were injected into a reversed-phase analytical column (Thermo Acclaim PepMap RSLC C18 column, 2 μm, 75 μm×50 mm) installed on an EASY-nLC UPLC instrument (Ultimate RSLCnano 3000). The peptides were eluted with the linear gradient of 2% to 10% buffer B (0.1% FA in 80% ACN) for 6 min, 10% to 20% for 45 min, and climbing to 80% within 4 min then holding at 80% for the last 1 min with the constant 250 nl/min flow rate.

The resulting peptides were subjected to a NanoSpray Ionization source followed by MS/MS in Q Exactive (Thermo Scientific) connected online to the UPLC system. The data-dependent acquisition mode was selected and the resolution for precursor ions detection was set at detected at a 60,000. The m/z mass scan range was 350–1800 Da for MS scan. For MS/MS analysis, the normalized collisional energy was set as 26 in HCD mode, and the resolution was 30000. The scan cycle was set as one MS scan followed by 15 MS/MS scans for the top 15 precursor ion acquisitions; the threshold for ion count in the MS survey scan was 1E5 with 6.0 s dynamic exclusion. The applied electrospray voltage was set to 2.0 kV, automatic gain control was used to prevent overfilling of the Orbitrap and 5E4 ions were accumulated for the generation of MS/MS. LC-MS/MS analysis was performed blindly by Micrometer Biotech Company (Hangzhou, China). All the MS data were deposited to ProteomeXchange Consortium via the PRIDE partner repository [21]. The accession number is PXD020788.

### Database searching

The resulted MS/MS data of 2-hydroxyisobutyrylated peptides was processed using MaxQuant software with integrated Andromeda search engine (v.1.4.1.2). Database for (*Triticum aestivum* L.) was obtained from Uniprot containing 130,673 sequences (released in October, 2019), which concatenated with reverse decoy database and common proteins contaminants. The detailed parameters were listed as follow. Mass error for precursor ions and precursor ions were set within 10 ppm and 0.02 D, respectively. Cleavage enzyme was specified as Trypsin/P with a maximum of 4 missing lysine or arginine sites. Fixed modification was defined as Carbamidomethylation on cysteine and variable modification was defined as oxidation on methionine and 2-hydroxyisobutyrylation on both lysine and N-terminal of protein. The false discovery rate for protein, peptide and modification site was specified at 1%. Peptide consisted of seven amino acid residues was set to the shorted peptide length for database search [17]. All of the other parameters were set to default.

### Bioinformatic analysis

Motif-x software was used for the motif analysis and secondary structures of proteins were predicted by NetSurfP [22]. Gene ontology (GO) analysis was derived from the UniProt-GOA database and the he further classification of the 2-hydroxyisobutyrylated proteins was performed based on the ontology of biological process, molecular function and cellular component [23]. Wolfpsort subcellular localization predication software was selected to perform the subcellular localization analysis [24]. The Kyoto Encyclopedia of Genes and Genomes (KEGG) database was used to annotate protein pathway [25] with Automatic Annotation Server (KAAS) tool. GO, KEGG pathway and domain enrichment analyses were carried out by DAVID tool [26]. For KEGG pathway enrichment analysis, the database for MS data searching was used as background while the identified Khib proteins were used as foreground. The pathways with over-represented Kac proteins were screened out. Correction for multiple hypothesis testing was carried out using standard FDR control method and annotation terms with a corrected p-value below 0.05 were considered as significantly enriched.

## Results and discussion

### Identification of Khib in wheat leaves

Wheat is an important staple food crop grown world-wide [27,28]. Previous studies have shown some PTMs such as Kac and Ksucc participate in various biological processes in wheat [29,30]. In the present study, to uncover the role of Khib, a novel discovered PTM, in wheat physiology and biology, a systematic qualitative lysine 2-hydroxyisobutyrylome was performed in wheat leaves. Previous studies have reported the global Khib analysis in developing rice seed and *Physcomitrella patens* haploid gametophyte [17,18]. Our study provided the first lysine 2-hydroxyisobutyrylome data of higher plant leave tissues, which enlarged the species and scope of Khib study in plant kingdom.

**Fig 1:**
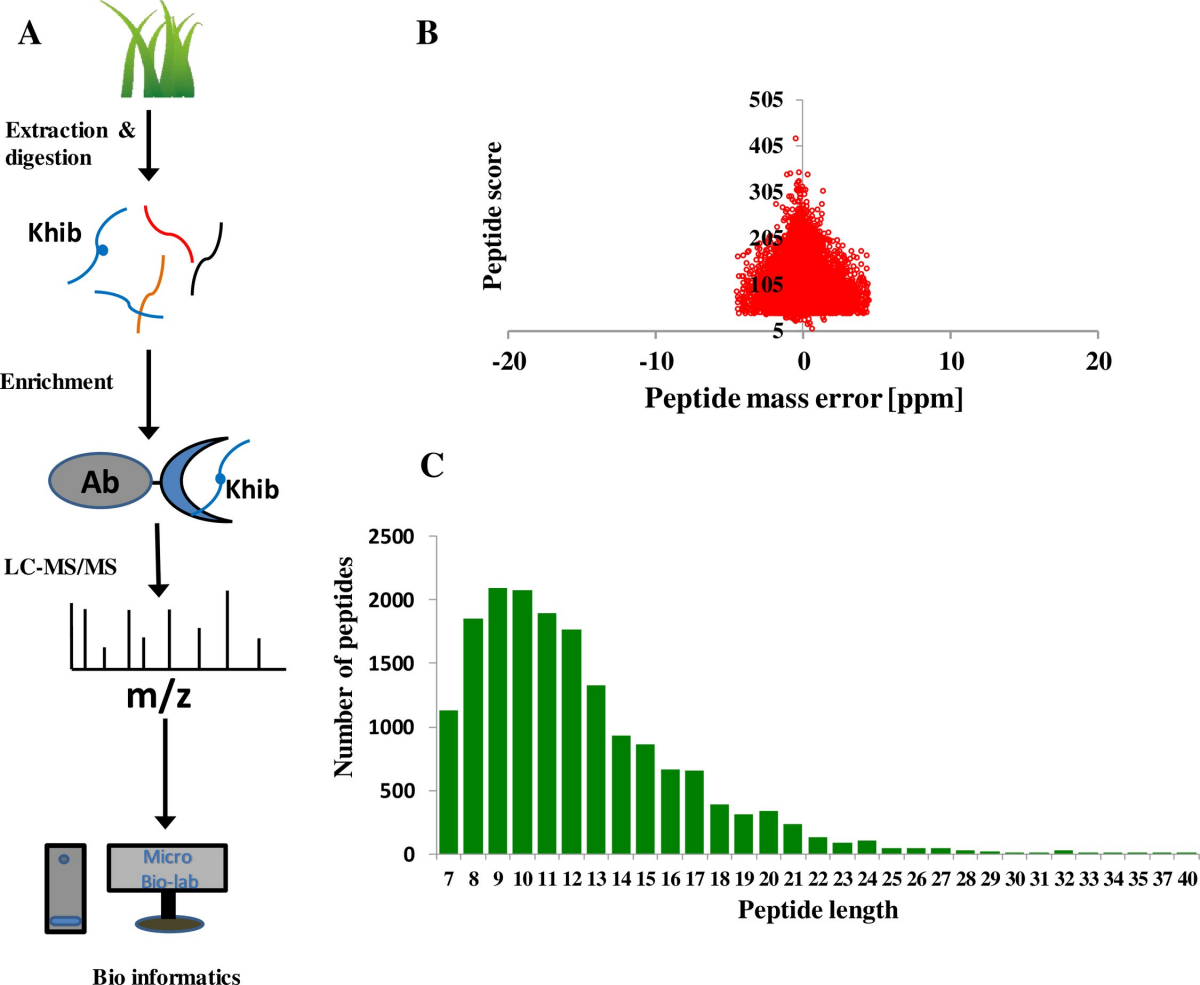
Identification of the global Khib sites and proteins in wheat leaves. (A) Workflow for global Khib detection in wheat leaves. (B) Mass error distribution of all the Khib peptides. (C) Peptide length distribution of all the Khib peptides.

The experiment design and procedures are shown in Fig 1A. In short, after wheat plant cultivation, wheat leaves were collected, and then the 2-hydroxyisobutyrylomic sample preparation procedures were performed including protein extraction, trypsin digestion and affinity enrichment for the modified peptides. Following was LC-MS/MS data acquisition and searching for the Khib peptides and proteins. Three biological replicate experiments were conducted and only the repeatedly identified Khib peptides and proteins were used for the bioinformatics analysis.

To insure the quality of the MS data, quality control analysis were performed (Fig 1B and 1C). The majority of peptides’ mass errors were within 5 ppm, indicating the high accuracy of MS data. As to the peptides length distribution, the most peptides’ lengths were located between 7 and 25 amino acids, showing the sufficient trypsin digestion. The MS data fits the requirement for further analyses.

**Fig 2:**
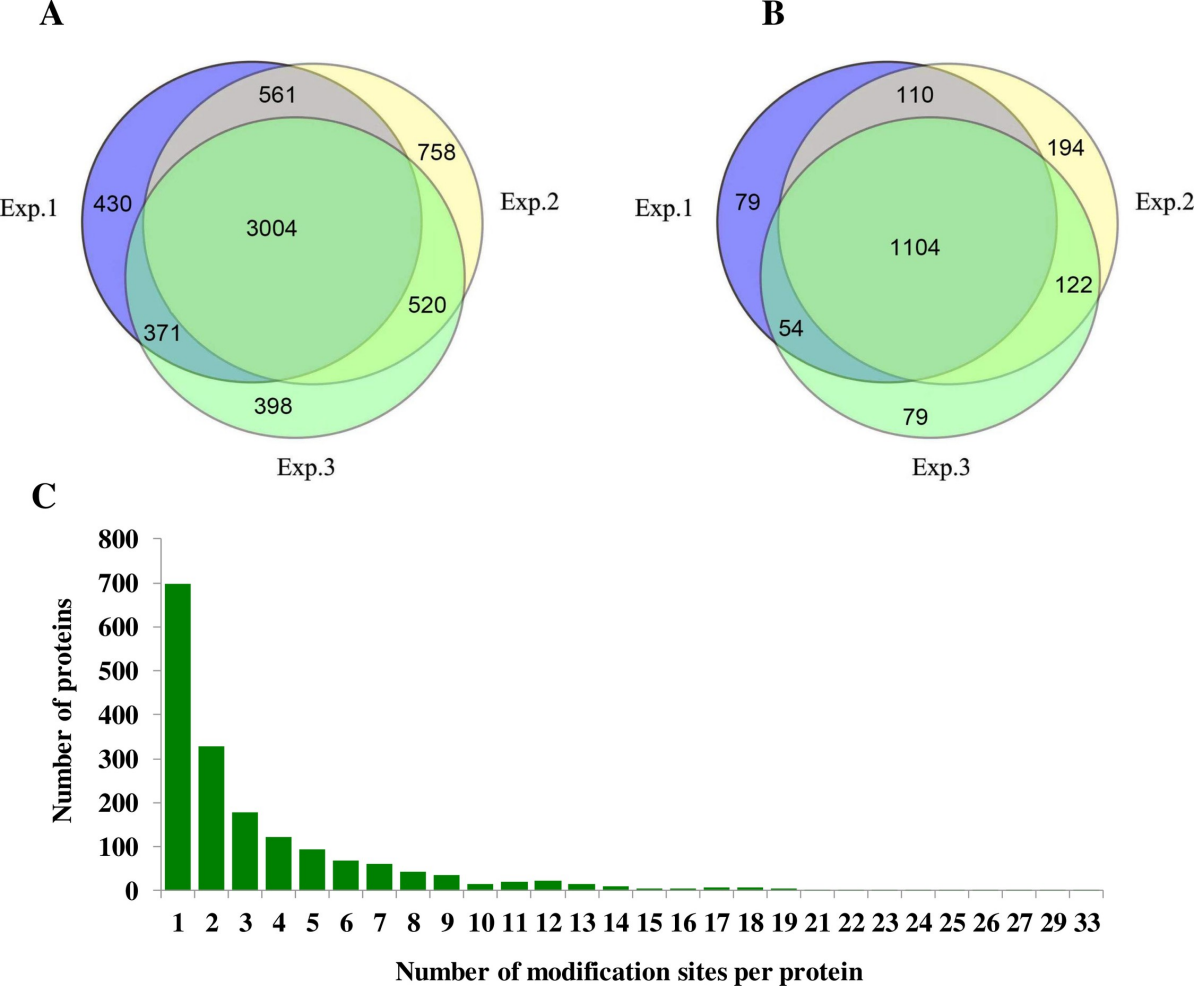
Statistics of the detected Khib sites and proteins. (A) Venn diagram of the detected Khib sites. (B) Venn diagram of the detected Khib proteins. (C) Number of Khib modified sites in a protein.

The detection results for the mentioned three replicates are provided in Fig 2 and S1 Table. Totally, 6042 Khib sites corresponding to 1742 proteins were detected, of which 3004 Khib sites corresponding to 1104 proteins were repeatedly identified; showing Khib was a widespread PTM in wheat (Fig 2A and 2B). The numbers of detected Khib sites on per protein were calculated to evaluate the frequency of Khib in wheat proteins (Fig 2C). The result indicated that 81.3% of proteins had 1–5 Khib sites, 12.5% contained 6–10 Khib sites. Only 6.2% had more than 10 Khib sites (Fig 2D).

### Site properties of the Khib peptides

**Fig 3:**
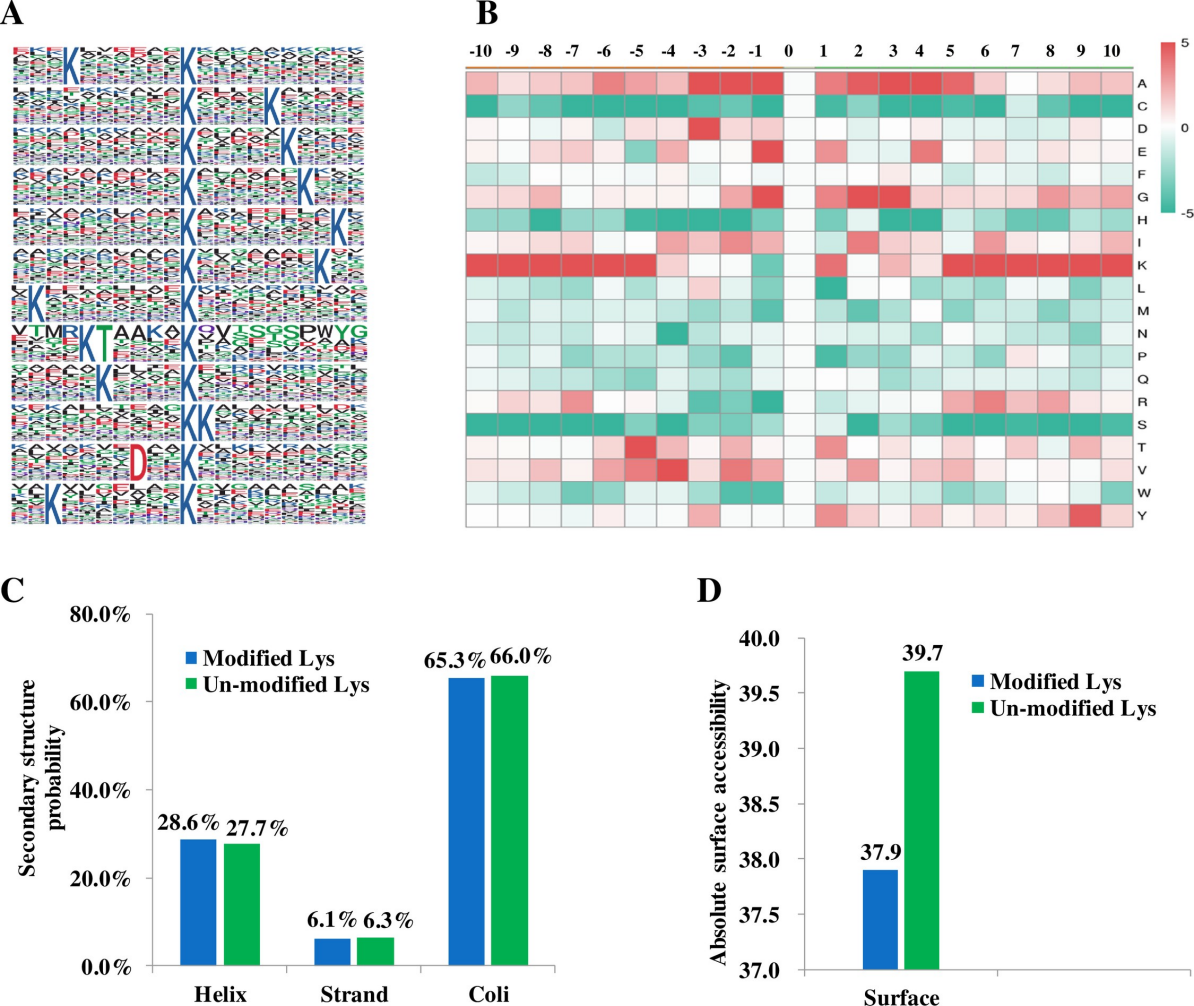
Structure characterizations of all the identified Khib peptides. (A) Motif analysis for amino acids around the Khib sites (−10 to +10). The letter height represents the frequency of that amino acid residue at that position. The K in the middle position corresponds to the Khib sites. (B) Heat map analysis of the amino acid compositions around the Khib sites. Red indicates an amino acid that is significantly enriched, while green indicates an amino acid that is significantly reduced. (C) Secondary structure analysis of Khib peptides and (D) Predicted surface accessibility of Khib peptides. Modified sites were marked in blue and unmodified sites were marked in green.

To show the features of the Khib peptides in wheat crop, motif analysis and amino acid heat map analysis were performed (Fig 3). Totally, 12 conserved Khib site motifs were extracted. Interestingly, as many as 10 lysine (K) containing motif were obtained, such as K******Khib, Khib****K, K****Khib and Khib******K (Khib indicates a 2-hydroxyisobutyrylation lysine, while * indicates a random amino acid residue, Fig 3A). Other defined conserved motifs included KT****Khib and D**Khib. Consistent with motif analysis result, the heap map analysis of the amino acid sequences surrounding the Khib sites indicated that K was over-represented in the −10 to −5 and +5 to +10 positions while under-represented in -4 to +4 positions (Fig 2B), which was highly similar with the sequence analyses of amino acids flanking the Khib sites in *Physcomitrella patens* haploid gametophyte [18]. In rice, it was also observed K appeared with relatively higher frequency in the −10 to −6 and + 6 to +10 positions surrounding the Khib sites [17]. Over-representation of K in the relatively far position and under-representation of K in the relatively near positions around Khib sites may be a shared sequence feature in different plant species.

Some amino acids with relatively small side chain groups including Alanine (A) and Glycine (G) were overrepresented at the very near positions (within 5 amino acids in both the upstream and the downstream) around the Khib sites. Previous study in *Physcomitrella paten*s haploid gametophyte indicated A were over-represented in all of the positions flanking Khib sites (-10 to +10) and G were over-represented in the majority of positions surrounding Khib sites [18]. In *Proteus mirabilis*, three A containing motif were extracted and A were drastically over-represented in -3, -1, +1 and +2 positions surrounding Khib sites [11]. It was noticed that the amino acids bearing relatively small side chain groups, such as G and A were welcomed in the sequence of Khib peptides. This phenomenon may be related to their better steric hindrance compatibility with 2-hydroxyisobutyrylation group as 2-hydroxyisobutyrylation was a relatively larger structural moiety (+86 Da) bringing significant steric hindrance effect [6].

To demonstrate whether Knib appeared frequently in certain space structure in wheat proteins, second structure analysis were carried out (Fig 3C and 3D). The result showed that 28.6% Khib sites were located in α-helix, 6.1% were located in β-strand and as high as 65.3% Khib sites were located in coil. In addition, surface accessibility evaluation indicated lysine residues at Khib sites lysine residues were relatively less surface-accessible compared with the unmodified lysine.

### Function classification and subcellular location analysis

**Fig 4:**
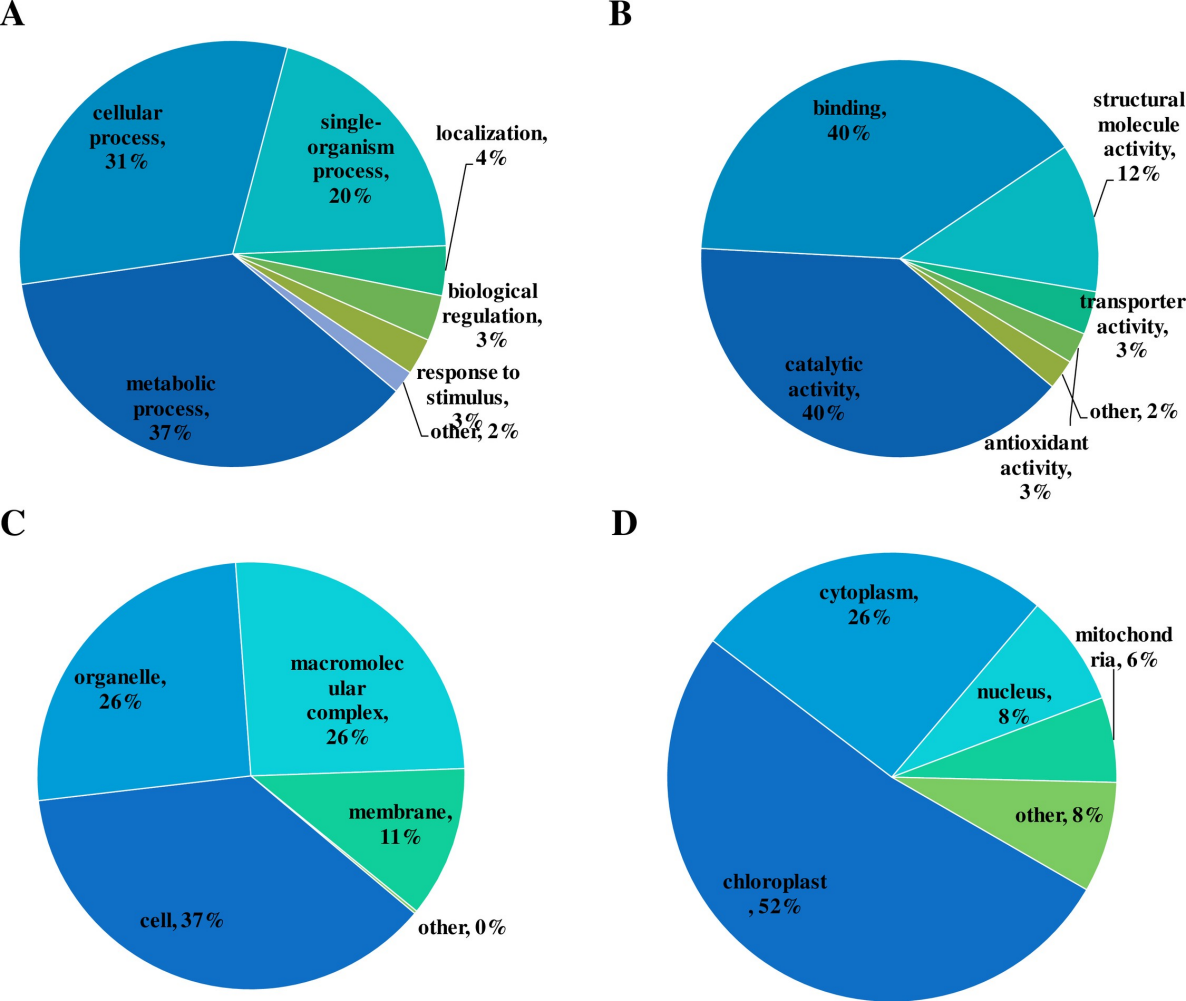
Function classification and subcellular localization of the detected Khib proteins. (A) Cellular component classification. (B) Biological process classification. (C) Molecular function classification. (D) Subcellular localization analysis.

Function classification and subcellular location prediction analysis were performed to decipher the nature of these Khib proteins (Fig 4). In the biological process level, the majority of these Khib proteins were metabolic process (37%), cellular process (31%) and single organism process (20%) related. In addition, some other biological processes including localization (4%), biological regulation (3%) and response to stimulus (3%) related were also observed while their proportion were relatively low. In the category of molecular function, the proportion of catalytic activity-related proteins (53%) and binding proteins (39%) were the top two highest, following is structural molecule activity (12%) related proteins. Transporter activity and antioxidant activity related proteins only accounted for 6% in sum. For the analysis of cellular component, diverse cellular components were involved, including cell (26), organelle (26%), macromolecular component (26) and membrane (11%).

Subcellular location analysis showed that more than a half (52%) Khib proteins were localized to the chloroplast (52%). The percentage of cytoplasm located proteins was the second highest (26%). Besides, 8% Khib protein and 6% Khib proteins were distributed at nucleus and mitochondria. Other subcellular locations related protein accounted for 8%.

The GO annotation based functional classification and WoLF PSORT-based subcellular location analysis indicated the Khib proteins involved in diverse biological processes and molecular events in wheat. In addition, in wheat leaves, protein Khib occurred in various cellular components and subcellular locations, of which chloroplast was the top subcellular compartment with 52% subcellular distribution proportion. The possible Khib influenced cellular events and biological processes within chloroplast should be deep explored.

### Enrichment analysis of Khib proteins

**Fig 5:**
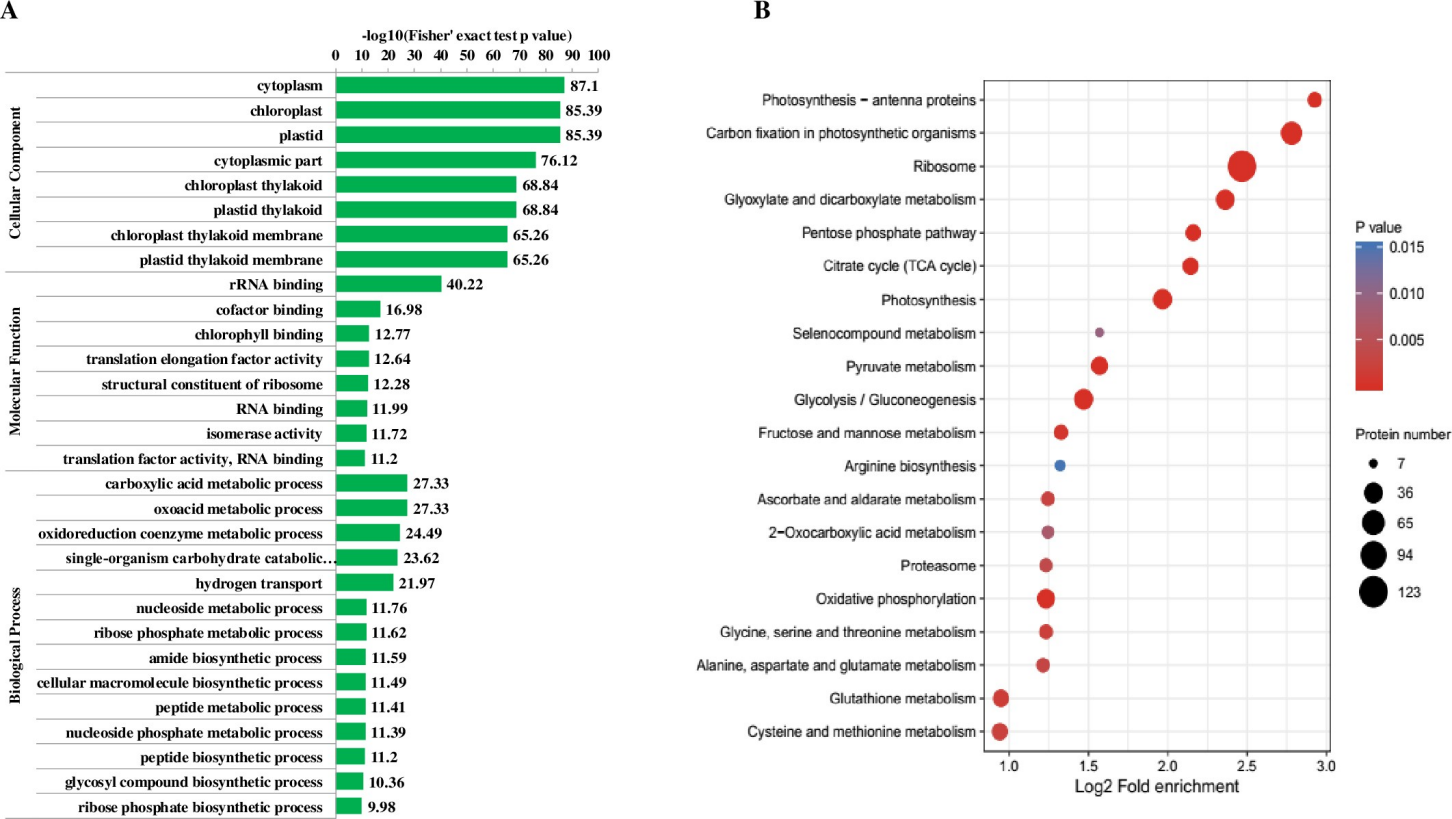
Enrichment analyses of all the Khib proteins. (A) Enrichment analysis based on GO annotation. (B) Enrichment analysis based on KEGG pathway.

To deeply uncover the characteristics of Khib proteins in wheat leaves, the GO enrichment, Kyoto Encyclopedia of Genes and Genomes (KEGG) pathway enrichment and domain enrichment analysis were conducted. As shown in Fig 5A, in the cellular component category, we noticed that all the significantly enriched components were cytoplasm or chloroplast/plastid related, which was consistent with the subcellular location prediction result. In the molecular function level, rRNA binding was the top significantly enriched term. In addition, the majority of the rest dramatically enriched terms were ribosome or translation related, suggesting the potential RNA translation and protein biosynthesis regulation of Khib in wheat. In biological process, a variety of metabolism related terms which involved many metabolic such as carboxylis acid, oxoacid, carbohydrate, hydrogen, nucleoside, ribose phosphate, amide and peptide etc., were observed, implying Khib in proteins probably influenced diverse metabolism activities.

Consistent with the biological process enrichment analysis, in KEGG pathway analysis, a number of metabolism pathways were significantly enriched (Fig 5B). Among these pathways, ribosome was dramatically enriched with the most Khib proteins. Moreover, numerous ribosomal proteins were Khib modified on multiple sites (S2 Table), indicating Khib may be an important regulation pattern of ribosome activity and proteins biosynthesis. Apart from ribosome, several photosynthesis related pathways were markedly enriched including photosynthesis-antenna proteins, carbon fixation in photosynthesis organisms and photosynthesis (Fig 5B), suggesting photosynthesis potentially could be affected by protein Khib in wheat leaves. The rest significantly enriched pathways showed a wide metabolism pathway distribution. Various carbon metabolism and energy production related pathways, such as citrate acid (TCA) cycle, pentose phosphate and pyruvate metabolism, glycolysis/gluconeogenesis and oxidative phosphorylation were markedly enriched. In addition, some amino acid metabolism related pathways were significantly enriched and the involved amino acids include arginine, glycine, serine, threonine, alanine, aspartate, glutamate and glutathione.

In the subcellular predication analysis, more than a half of Khib proteins were localized to chloroplast (Fig 4D), imply the biological activities and metabolism pathways within chloroplast may be the major targets which Khib influenced. A KEGG pathway enrichment analysis of all the chloroplast located proteins was performed to demonstrate the potential Khib regulated biology and metabolism pathways in chloroplast (S1 Fig and S3 Table). As the result shown, ribosome was still the top dramatically enriched pathway with the largest Khib protein numbers, indicating protein biosynthesis within chloroplast may be actively regulated by Khib. Besides, as expected, three photosynthesis related pathways were markedly enriched, including photosynthesis-antenna proteins, carbon fixation in photosynthesis organisms and photosynthesis, which fit to the primary intrinsic biological role (photosynthesis) of chloroplast in plant.

In the domain analysis (S2 Fig) of all the Khib proteins, chlorophyll a/b binding protein domain was the most significantly enriched domain, which was consistent with the markedly enriched photosynthesis antenna proteins in the pathway analysis (Fig 5B). In accordance with the dramatically enriched ribosome in pathway analysis and the significantly enriched ribosome and translation related molecular function terms, some ribosome and translation protein related domains were observed in the domain enrichment analysis, such as translation protein SH3-like domain and beta-barrel domain, ribosomal protein L2 domain 2 and S5 domain 2-type fold. Other significantly enriched domains were mainly related to enzymes and proteins belonging to glycometabolism and energy production processes, such as lactate/malate dehydrogenase, oxidoreductase, glyceraldehyde 3-phosphate dehydrogenase (GAPDH), FAD/NAD-linked reductase and electron transport accessory protein.

### Khib influenced ribosome activity and protein biosynthesis in wheat leaves

Previous study in yeast has shown the Khib proteins were strongly enriched to the ribosome pathway and aminoacyl-tRNA biosynthesis pathway [10]. In A549 cells, it was reported up-regulated Khib proteins were dramatically enriched to ribosome [14]. Significantly enriched ribosome pathway and aminoacyl-tRNA biosynthesis pathway were also observed in developing rice seed tissues [17]. The Khib modification on ribosome related proteins may be a common phenomenon in various species and tissues and it potentially regulated ribosome structure and activities and further influenced gene translation and protein biosynthesis in cell. Our findings (Figs 5 and S2) indicated the Khib may also play a ribosome activity regulation and protein synthesis control role in wheat leaves. In addition, the KEGG pathway analysis of all the chloroplast located proteins (S1 Fig and S3 Table) indicated Khib influenced ribosome activity may be an active regulation pattern of proteins synthesis within chloroplast. Protein synthesis in chloroplast serve for the translation of their own tiny genomes (60–100 coding gene) and the resulted products usually form central complexes of many crucial biological processes such as photosynthesis, metabolism and gene expression, together with nucleus-derived subunits [31–33]. Khib modification on both chloroplast located ribosome and non-chloroplast located proteins may affect the formation of these central complexes through controlling the biosynthesis of their subunits and components, and consequently regulate various biological processes and cellular events in leave tissues apart from directly regulating the functions and activities of the proteins or enzymes in certain pathways.

### Khib regulated photosynthesis in wheat leaves

Photosynthesis is of vital importance to plant and even to the all lives on the earth as it is the most extensive biosynthesis process in nature which transfers the energy of the sun to chemical energy and reduces carbon dioxide (CO2) to oxide oxygen [34,35]. The dramatically enriched photosynthesis related pathways (Fig 5B) indicated the photosynthesis processes including both light reaction and dark reaction in wheat may be influenced by Khib.

**Fig 6:**
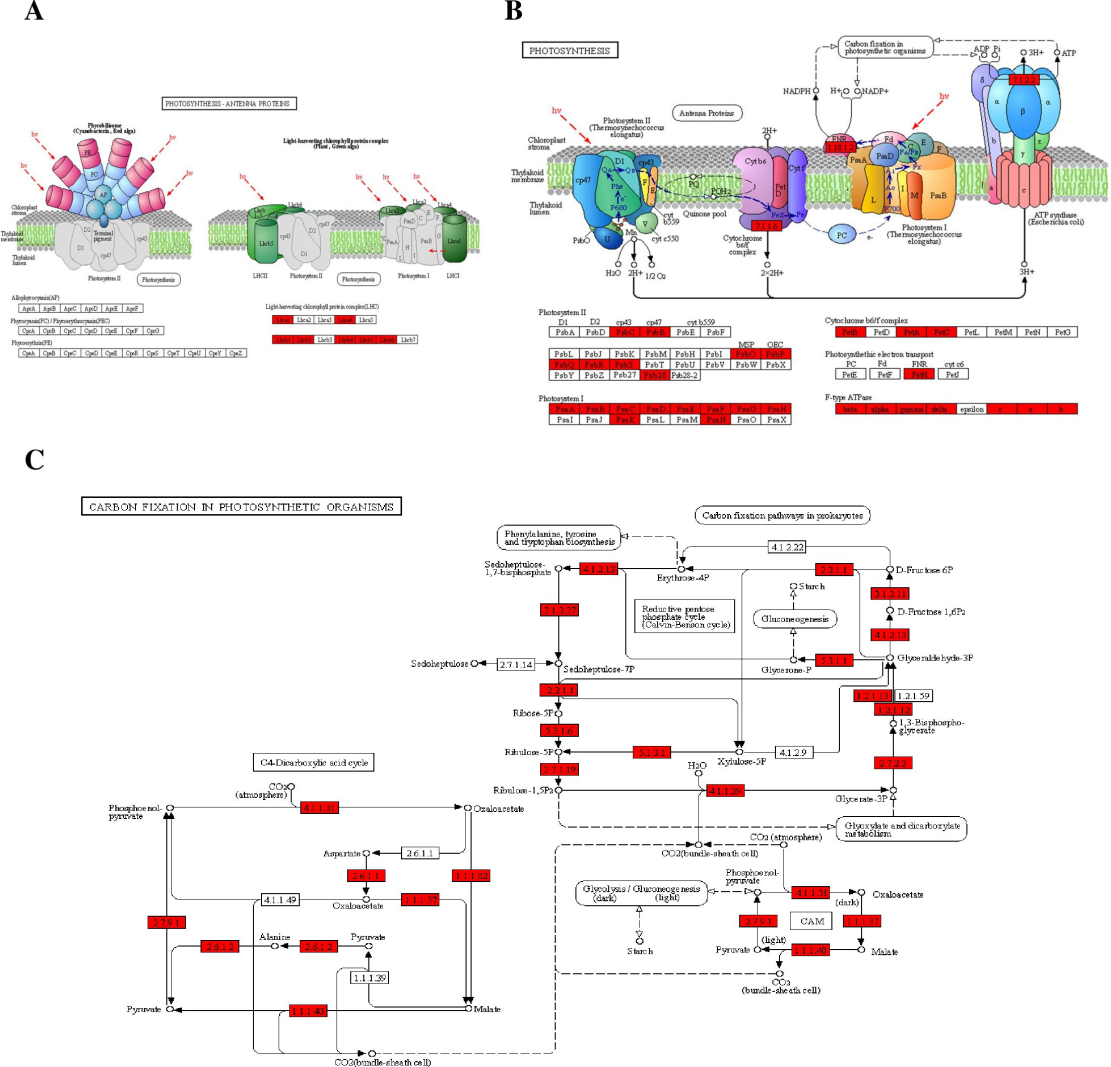
Representative significantly enriched photosynthesis related KEGG pathways. (A) Photosynthesis-Antenna proteins. (B) Photosynthesis. (C) Carbon fixation in photosynthetic organisms. The Khib proteins were labeled in red.

The representative significantly photosynthesis related pathways including photosynthesis-antenna proteins, photosynthesis and carbon fixation in photosynthesis organisms were shown in Fig 6. As Fig 6A and 6B indicated, all the core complexes and members (photosystem I and photosystem II, cytochrome b6f complex, ATP synthase and electron transport chain) in the light reaction were Khib modified on a large number of subunits or components of these macromolecular complexes. Chlorophyll a/b-binding proteins are the dominant components of light harvesting complex/antenna complex (LHC), which function as a light energy capture and transfer role in light reaction [36]. As much as 23 Khib sites were identified on chlorophyll a/b-binding proteins (S2 Table), which was consistent with the most significantly enriched protein domain (Chlorophyll a/b binding protein domain) in the domain analysis (S2 Fig). The potential influences of Khib on light harvesting and transferring need to be further studied. Previous study in the relatively lower plant *Physcomitrella patens* have found some Khib sites and proteins in light reaction processes [18]. Our study in the relatively higher plant wheat expanded the scope and enlarged the datasets of photosynthesis light reaction related Khib sites and proteins, which is of potential study significance in photosynthesis light reaction efficiency elevation.

The enzymes participating photosynthesis dark reaction (Calvin cycle) also exhibited multiple Khib sites (Fig 6C and S2 Table), implying carbon fixation process potentially be influenced by Khib modification. The involved enzymes included ribulose bisphosphate carboxylase (Rubisco), fructose-bisphosphate aldolase, malate dehydrogenase, fructose-bisphosphate aldolase, glyceraldehyde-3-phosphate dehydrogenase, triosephosphate-isomerase, fructose-1,6-bisphosphatase phosphoglycerate kinase, phosphoribulokinase, phosphoenolpyruvate carboxylase 1, and ribulose-phosphate 3-epimerase, etc. Among these enzymes, Rubisco, the key enzyme of carbon assimilation exhibited the most Khib sites with 22 Khib sites in the Rubisco large subunit (RBCL) and 5 Khib sites on the Rubisco small subunit (RBCS). Rubisco catalyze the first step of CO_2_ assimilation, which is the rate-limiting step of photosynthesis in plant [37]. The Khib sites on the RBCL and RBCS may adjust the interactions among these subunits through changing the charge states and steric hindrances of subunit interaction interface; as well as forming hydrogen bonds inter subunits [6,9]. Then the catalyzing activity of intact Rubisco complex is influenced. The high proportion catalyzing and binding related protein in the function classification analysis in molecular function level supported this view (Fig 4B). In addition, some potential Khib modification may occur nearby the catalyzing domain, which could directly participate in catalyzing activity. Further structure biology study is needed to verify this assumption. Because of the critical role of Rubisco in photosynthesis, it is often regarded as a potential molecular target of genetic engineering for the agriculture plant yield increasing purpose [38]. Adjusting the Khib modification level of Rubisco through gene engineering techniques may be an approach which is worth to explore.

### Overlap among Khib, Kac and Ksuc in wheat leaves

Previous studies have shown there was crosstalk among various PTMs and some PTMs coexist on a single protein molecule, especially on histones [39,40]. These multiple PTMs on function proteins show the crosstalk effects in diverse patterns for the better biological process and metabolism pathway regulation purpose [40,41].

**Fig 7:**
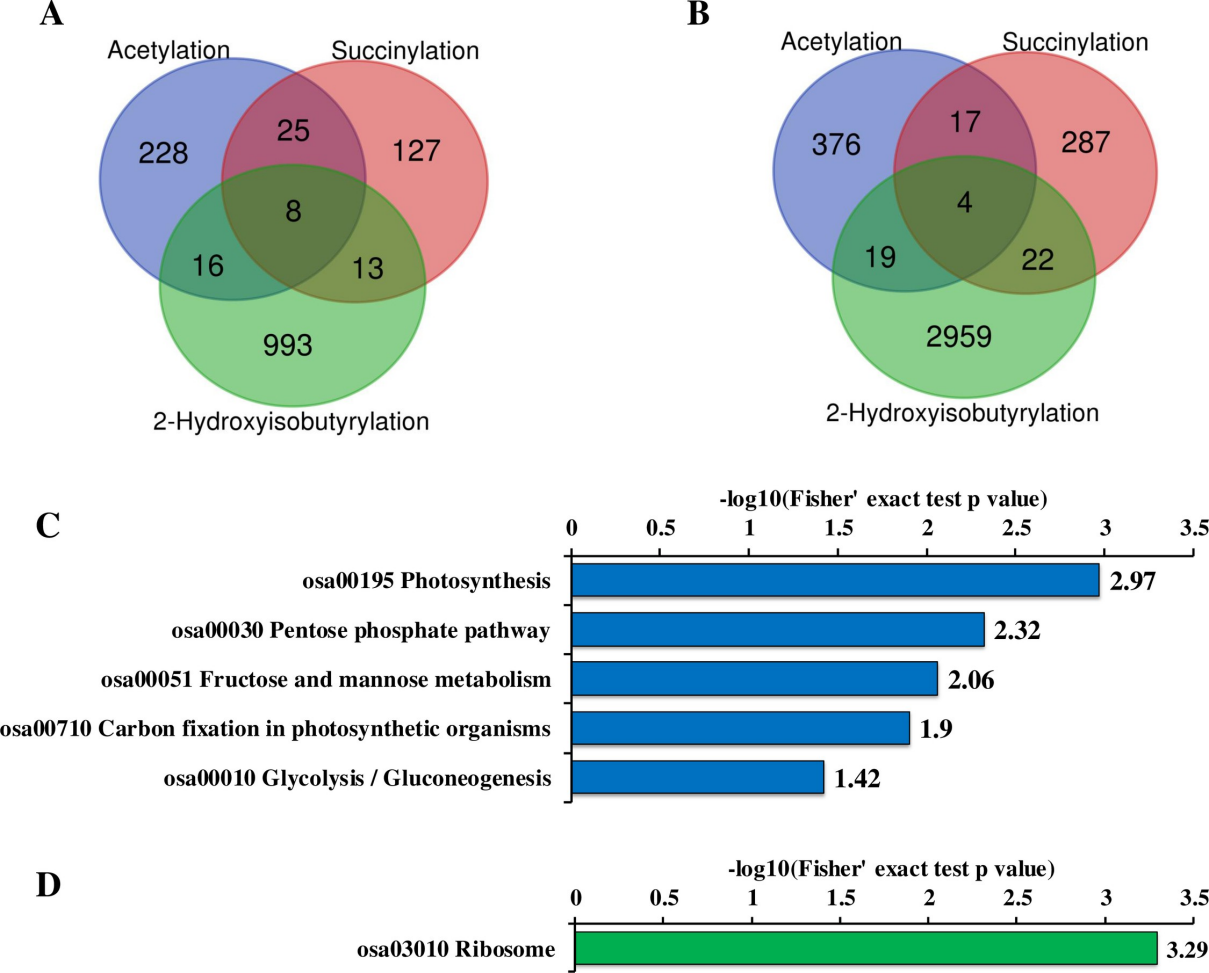
Overlap analyses among wheat acetylome, succinylome and 2-hydroxyisobutyrylome. (A) Overlap of Kac, Ksucc and Khib proteins. (B) Overlap of Kac, Ksucc and Khib sites. (C) KEGG pathways enrichment of the overlapped proteins between Kac and Khib. (D) KEGG pathways enrichment of the overlapped proteins between Ksucc and Khib.

The previous Kacand Ksucc analysis at proteomics level in wheat leaves have shown there were some overlapped proteins and even lysine sites between acetylome data and succinylome data [29,30]. We further performed the Venn diagram analysis among Khib, Kac and Ksucc modifications by comparing our Khib dataset with published wheat acetylome and succinylome datasets, to uncover whether Khib harbor some common proteins and lysine sites with Kac and/or Ksucc. As shown in Fig 7A and S3 Table, 24 proteins were modified by both Khib and Kac, and 21 proteins harbored Khib and Ksucc modification. Moreover, 8 proteins encompassed all the three PTM. The overlapped sites screening result (Fig 7B and S4 Table) indicated Khib shared 23 lysine sites with Kac and 26 lysine sites with Ksucc. In addition, 4 lysine sites exhibited these three PTMs. Previous studies showed that different PTMs at the same proteins or the same sites may have potential functional cross-talk [42,43]. We speculate the functions or activities of these proteins bearing Khib modification and Kac/Ksucc modification may be influenced by the crosstalk effects among these PTMs. KEGG pathways enrichment analysis were performed to reveal the potential metabolism pathways influenced by these co-modifications. As the result shown, Khib and Kac crosstalk probably interact and regulate some photosynthesis and carbohydrates metabolism related events in wheat leaves as Khib and Kac co-modified proteins were significantly enriched into photosynthesis and glycometabolism related pathways (Fig 7C). The proteins suffered both Khib modification and Ksucc modification were markedly enriched to ribosome (Fig 7D), implying ribosome activity and protein biosynthesis process in wheat leaves may be co-regulated by Khib and Ksucc. Further investigation was required to validate these crosstalk and uncover the underling interplay mechanisms.

## Conclusion

In this wok, the first wheat lysine 2-hydroxyisobutyrylome analysis was conducted with leave tissues. Totally, 3004 Khib sites corresponding to 1104 proteins were repeatedly identified, indicating Khib was a widespread PTM in wheat. Motif analysis extracted 12 conserved sequence motifs and heap map analysis suggested K, G, A were relatively active amino acids surrounding the Khib sites. These Khib proteins showed a wide biological process and molecular function distribution. Moreover, chloroplast is the primary subcellular compartment where Khib are distributed. Deeper enrichment analysis illustrated the ribosome activity and protein biosynthesis both in chloroplast and out chloroplast may be regulated by Khib modification. In addition, photosynthesis including both light reaction and dark reaction processes potentially is influenced by Khib. The co-occurrence of Khib, Kac and Ksucc were observed in some proteins, implying the potential crosstalk and function interaction in these PTMs. Our study expanded the scope of Khib in plant species, which could be used as a resource and reference of Khib function demonstration and structure characterization in cereal plant, as well as in plant kingdom.

## Supporting information

S1 Fig Domain enrichment analysis of all the identified Khib proteins. (PDF)

S2 Fig KEGG enrichment analysis of all the chloroplast located Khib proteins. (PDF)

S1 Table Summary of all the identified Khib sites and proteins in wheat leaves in three replicates. (XLSX)

S2 Table The detailed information of the significantly enriched KEGG pathways from all the identified Khib proteins. (XLSX)

S3 Table The detailed information of the significantly enriched KEGG pathways from all the chloroplast located Khib proteins. (XLSX)

S4 Table The list of the all the overlapped modified proteins among wheat acetylome, succinylome and 2-hydroxyisobutyrylome. (XLSX)

S5 Table The list of all the overlapped modified lysine sites among wheat acetylome, succinylome and 2-hydroxyisobutyrylome. (XLSX)
