# Supplementary figures and images for: Systematic analysis of lysine 2-hydroxyisobutyrylation posttranslational modification in wheat leaves
Source: PLoS One. 2021 Jun 17;16(6):e0253325. doi: 10.1371/journal.pone.0253325 (PMC8211214; doi:10.1371/journal.pone.0253325)

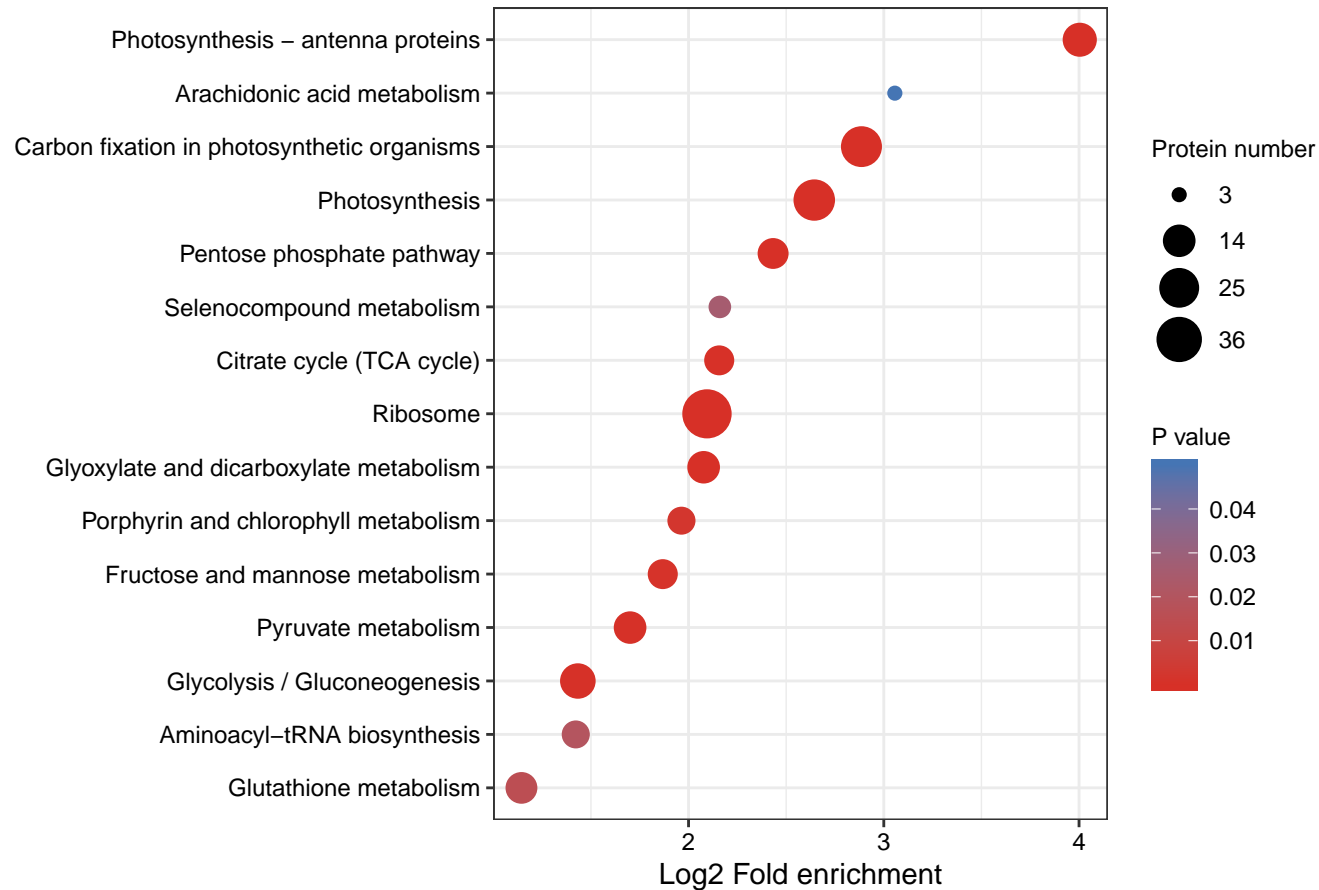

Supplement: S1 Fig — (PDF) [file pone.0253325.s001.pdf]

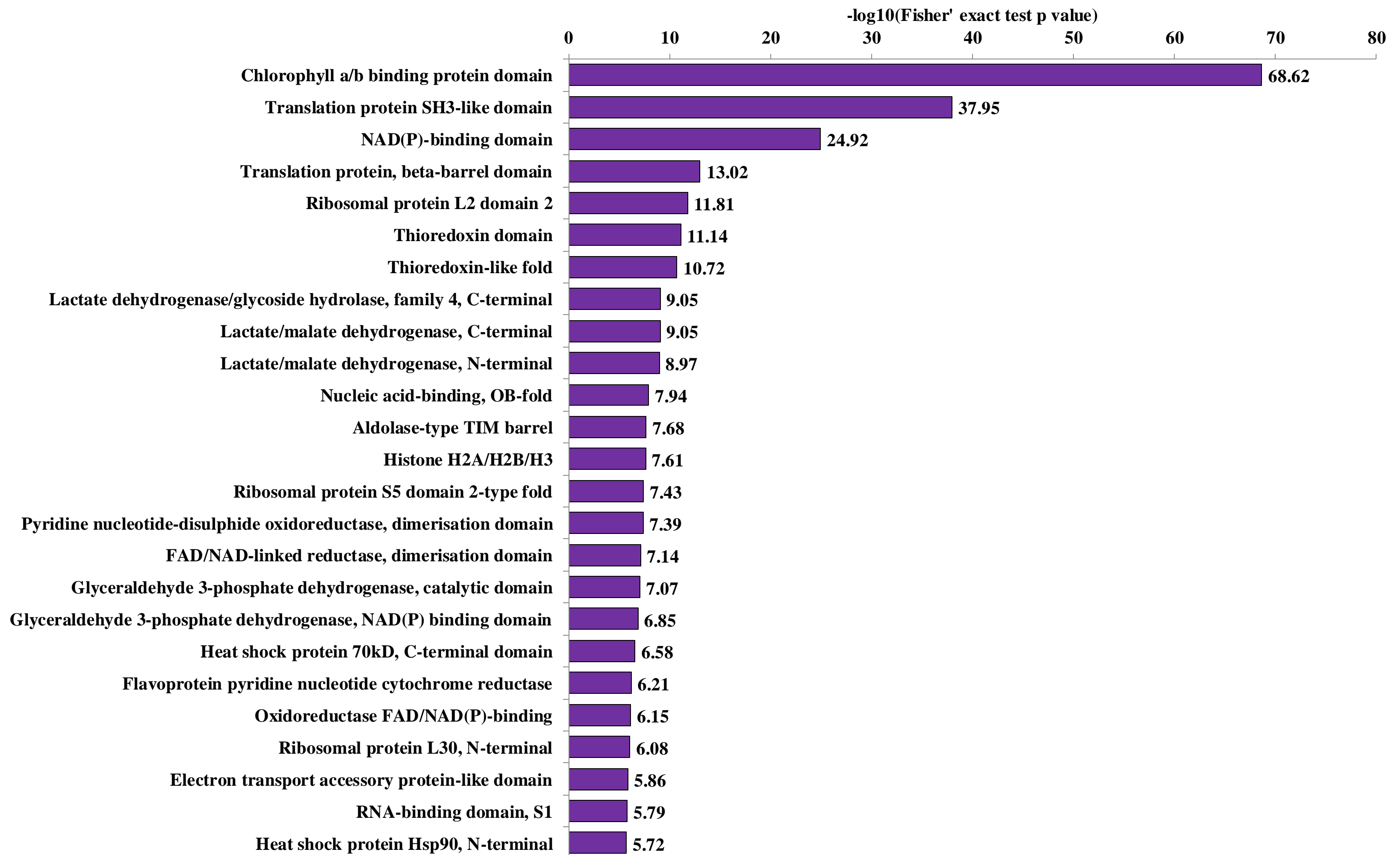

Supplement: S2 Fig — (PDF) [file pone.0253325.s002.pdf]
